# Human Papillomavirus-Associated Cancers and the Potential Role of Local Bacterial Populations

**DOI:** 10.3390/cancers18213420

**Published:** 2026-10-22

**Authors:** Amitabha Ray, Daniel M. Borsch, Rajashree Pandit

**Affiliations:** 1 Basic Sciences, Ponce Health Sciences University, Tortola VG1110, British Virgin Islands; 2 Department of Biology, The Community College of Allegheny County-Allegheny Campus, Pittsburgh, PA 15212, USA; dborsch@ccac.edu; 3 School of Public Health, Boston University, Boston, MA 02118, USA; rajmph@bu.edu

**Keywords:** HPV genome, cervical cancer, oropharyngeal cancer, *Lactobacillus* species, anaerobic bacteria, dysbiosis, gut microbiota, probiotics

## Abstract

Human papillomavirus (HPV) is the most common sexually transmitted infectious agent in the United States as well as globally. This review aims to examine neoplastic diseases associated with HPV with special emphasis on cancers of the uterine cervix and oropharynx. Among the group of high-risk HPVs, i.e., types of HPV that can cause abnormal cellular changes leading to malignancies, HPV genotypes 16 and 18 are commonly found in cervical cancer, whereas HPV-16 is the most detected HPV in oropharyngeal cancer. It is known that the HPV genome comprises three major functional regions: the early region, which encodes proteins such as E6 and E7; the long control region; and the late region, which encodes structural capsid proteins L1 and L2. The E6 and E7 proteins are pivotal to carcinogenesis. E6 promotes degradation of the tumor suppressor p53, while E7 inactivates the retinoblastoma protein, critically disrupting cell cycle regulation and enabling uncontrolled proliferation. These disruptions promote survival of HPV-infected cells–a requirement for the viral life cycle. The viral L1, the major capsid component, is used for classification, PCR detection, antigenic tests, and in prophylactic vaccine preparation. However, it is important to correlate the role of HPV with different risk factors, particularly when considering a wide variation in cervical cancer incidence rates in different nations and geographic regions. Compared to cervical cancer, HPV-positive oropharyngeal cancer risk factors are thought to be associated with sexual behavior primarily. Overall, the local microbial environment of both the cervicovaginal region and the oral cavity may play a significant role in cancer development. For example, an increase in certain bacteria has been documented in the course of disease, such as *Fusobacterium* in oropharyngeal cancer or *Prevotella* and *Gardnerella* in bacterial vaginosis. On the other hand, a depletion of beneficial bacteria in the cervicovaginal environment may contribute to the development of cervical neoplasia. In the case of cervical cancer, all risk factors possibly alter the cervicovaginal microbial flora adversely. The dominance of anaerobic bacteria could bring about pathological changes. In relation to the disease progression, an increasing level of *Lactobacillus* depletion has been reported. Similarly, in different head and neck cancers, investigators documented differences in bacterial genera between HPV-positive and HPV-negative cases. Furthermore, some investigators suggested that certain bacteria could serve as cancer biomarkers in oral regions. Various bacteria are present throughout the gastrointestinal tract, particularly in the large intestine (colon), where they form the largest populations, and they influence diverse pathophysiological processes both inside and outside the gut. The gut microbiota is a typical example of how alterations in it could be associated with pathological processes, including cancer development. Regarding their impact on other organs, different bidirectional communication networks have been proposed. One pertinent example is the gut–vaginal axis, which connects the microbial communities of these two organs. Currently, a growing number of studies are trying to manipulate this axis and reconstitute the cervicovaginal microbial flora in a favorable manner. Therefore, appropriate modifications of the local microbial environment could prevent pathological processes in an economical way.

## Simple Summary

Only a few cancers have been classically linked with human papillomavirus (HPV), although HPV has been isolated in tumor tissues from several sites. Nevertheless, the two most common HPV-related malignancies are cancer of the uterine cervix and cancer of the oropharynx, which includes the palatine and lingual tonsils, among the heterogeneous group of head and neck cancers. The first part of this review describes the epidemiology, pathological processes, and relevant diagnostic approaches for both cervical and oropharyngeal cancers in a conventional manner. In the latter part, mainly bacterial issues have been discussed, with a particular focus on cervical cancer. Perhaps, all the risk factors for cervical cancer ultimately disturb or negatively alter a healthy cervicovaginal microbial composition. On the other hand, a growing body of evidence suggests that HPV alone likely does not cause malignant transformation; other risk factors such as smoking and lack of beneficial bacteria in the cervicovaginal environment possibly contribute to pathogenesis. This review examines findings, though preliminary, from relevant studies and highlights a potential preventive strategy of using probiotics that is affordable for the masses.

## 1. Introduction

As per the report of the Centers for Disease Control and Prevention (CDC), infection with the human papillomavirus (HPV) is the most common sexually transmitted disease in the United States. This DNA virus, which has a preference for squamous epithelium, can cause common warts, genital warts, and cancer. According to the World Health Organization (WHO), at least 13 HPV genotypes are linked with cancer in different sites, such as the uterine cervix (cervical cancer), the anogenital region, as well as the head and neck (particularly the oropharynx). Approximately 30% of infection-linked cancers (i.e., 5% of all cancers globally) are associated with HPV [1,2]. It is worth mentioning that 20–25% of all cancers are connected with various infectious etiologies [1]. With regard to cervical cancer, HPV is responsible for more than 80% to about 90% of cases, and a majority of these cases (more than two-thirds) are from low- and middle-income countries [3,4]. In addition, among HPV-attributable cancers, the relative contribution of HPV-16 and -18 is about 70% [2,3]. Of note, HPV-16 is usually the most commonly isolated high-risk HPV type in cancer.

After the introduction of HPV vaccination in 2006, various investigators have noticed a reduction in certain HPVs, particularly HPV-16 [5,6]. However, many studies that may not have been related to the vaccine effect also showed changes in HPV genotypes. For example, Dong et al., during their screening from 2005 to 2014, observed that HPV-16 and HPV-52 were the most common subtypes in the population, and HPV-16 prevalence decreased over time [7]. In a cross-sectional study of 10,104 women from 2019 to 2022, Chen et al. reported that the most frequently isolated subtypes were HPV-52, HPV-16, HPV-58, HPV-51, HPV-53, and HPV-66 [8]. In general, the post-vaccination period displays continued replacement of common HPV types such as 16, 18, and 31 by others such as HPV-35, 39, 51, 52, 56, 58, 59, and 66 [6]. In a study in Spain, Freire-Salinas and colleagues observed that HPV-31, HPV-52, and HPV-45 increased in percentage, but the decrease in HPV-16 was non-significant [5]. In addition, regarding the protective mechanisms, they highlighted the phenomenon of ‘herd effect’, which indicates indirect protection for unvaccinated individuals due to a diminished prevalence of HPV among the population. Nevertheless, future screening and vaccination strategies need to consider this genotype-changing trend.

During the long evolutionary process of human development, HPV is believed to have existed in the body since archaic humans (these transitional hominins were present approximately from 600,000 to 30,000 years ago) [9]. Probably, due to HPV’s co-evolution with humans for an extended period, infections are generally asymptomatic [10]. Nevertheless, HPVs are categorized into five main genera, i.e., alpha, beta, gamma, mu, and nu [11]. Each genus is further classified into different species, and each species includes several HPV types. For example, the species group alpha-9 comprises HPV-16, along with HPV-31, HPV-33, HPV-35, HPV-52, HPV-58 and HPV-67 [12]. On the other hand, the alpha-7 species group contains HPV-18. Usually, the alpha genus includes pathological HPVs–both high-risk or carcinogenic types and low-risk types that generally cause benign lesions like warts. HPVs are relatively small, non-enveloped viruses with double-stranded circular DNA of around 8 kb. The genome has three main parts: (i) an early region that encodes the E1, E2, E4, E5, E6, and E7 proteins, which are associated with gene expression, replication, and survival, (ii) a long control region (LCR) that regulates DNA replication and transcription, and (iii) a late region, which encodes the structural or capsid proteins L1 and L2 [11].

It is well known that the viral E6 and E7 proteins are primarily responsible for carcinogenesis because they cause the degradation of p53 and retinoblastoma protein (pRb), respectively. Both p53 and pRb are tumor suppressor proteins that regulate the normal cell cycle and proliferation and eliminate defective cells. However, the survival of the virus-infected defective cells is necessary for the continuation of the viral life cycle, but it increases the risk of cancer. Recently, in connection with E6 and E7, several other cancer-related mechanisms have been proposed, such as histone modification, modulation of the microRNA network, and induction of the epithelial-to-mesenchymal transition (EMT) [13]. Additionally, viral E5 is believed to promote cell proliferation chiefly through epidermal growth factor receptor (EGFR) signaling. On the other hand, the HPV E4 protein, which exists as the E1^E4 form (fusion protein resulting from the initial sequence of amino acids from the E1 open reading frame due to mRNA splicing), is thought to be linked with viral release by disrupting epithelial intermediate filaments [14,15].

All documented papillomaviruses encode a class of core proteins, which existed early in the course of evolution and include E1, E2, L1, and L2 [10,16]. In viral transcription and replication, both the E1 and E2 proteins play a crucial role. The icosahedral capsid of the virus is formed by the major capsid protein L1 (360 copies) and the minor capsid protein L2 (up to 72 copies), and these two proteins are produced in terminally differentiated cells [17,18]. L1 forms pentameric monomers along with L2, which contain 5 L1 and 1 L2 proteins, that eventually assemble into 72 capsomeres [19]. In addition, the L1 protein comprises both constant and variable regions; the variable region contains surface-specific antigenic epitopes. HPV L1 possesses a number of crucial qualities. For example, to initiate HPV infection, the L1 protein performs the primary role–its positively charged lysine residues first interact with the negatively charged heparan sulfate proteoglycans present on the epithelial surface [15,18]. HPVs are classified based on the similarity or divergence of their L1 gene sequences; for instance, a nucleotide pairwise identity (or percentage of identical nucleotide bases) of more than 90% must be met to be considered the same type, and more than 60% to be in the same genus [10]. In the laboratory, for the detection of HPV DNA, the PCR method is applied using L1 consensus primers–for instance, MY09/MY11, located within the conserved L1 region of the HPV genome. In the immunohistochemical detection of HPV, antibodies against the L1 protein are commonly used. Furthermore, L1 proteins can self-assemble into capsid-like arrangements, known as virus-like particles (VLPs), which are currently used in HPV vaccines. Upon vaccination, neutralizing antibodies are finally produced against the L1 protein, which block HPV from infecting susceptible cells [20]. It has been reported that the HPV vaccine efficacy is not similar in different HPV-related cancers [2,21,22]. Vaccine efficacy is better in cervical cancer compared to other HPV-related cancers, including head and neck cancers. In general, vaccine efficacy is assessed by evaluating disease development in vaccinated individuals as opposed to controls in a controlled clinical trial. Contrarily, vaccine effectiveness denotes how a vaccine works in various populations.

In this review, an attempt has been made to examine two significant HPV-related neoplastic diseases: cervical cancer and head and neck cancers (mainly oropharyngeal cancer), with a particular focus on cervical cancer and the important role of the cervicovaginal bacterial population. Overall, their epidemiology, pathogenesis, and laboratory diagnoses are helpful for a comprehensive understanding of the disease processes. Appropriate research findings, including other relevant articles, were collected from MEDLINE/PubMed (mainly) and Google Scholar. Specifically, for the last two Tables, only recently published studies available in PubMed were considered and obtained by using suitable keywords such as “head and neck cancer”, “HPV” and “bacteria”; “oropharyngeal cancer” and “bacteria”; “cervical cancer” and “bacteria”; and “vaginal bacteria” and “probiotics”. Overall, the aim was to collect the recent literature (published in the last 5 years), which was subsequently assessed manually for suitability.

## 2. HPV-Related Oropharyngeal Cancer and Comparison with Cervical Cancer

According to the WHO’s recent reports about the global incidence rates (including all cancers), cervical cancer is the 8th most common cancer; and among women, it is the 4th most common cancer (roughly 660,000 new cases and 350,000 deaths worldwide). In contrast, head and neck cancer is the 6th most common cancer worldwide [23]. Nearly 90% of head and neck cancers are squamous cell carcinoma [24]. According to Barsouk and his colleagues, head and neck squamous cell carcinoma (HNSCC), which includes cancers of different sites, e.g., oral cavity, pharynx, larynx, salivary glands, and nasal cavity, constitutes the 7th most common cancer globally [25]. Oral cavity cancer sites include the lips, gums, anterior two-thirds of the tongue, floor of the mouth, buccal mucosa, and hard palate. Considering anatomical sites and HPV status, HNSCC can be divided into two distinct cancer groups. For example, HPV-positive HNSCCs typically occur in the oropharynx, which is the area located posterior to the oral cavity, encompassing the tonsils (palatine and lingual tonsils), the base of the tongue (where the lingual tonsils are situated), and the soft palate. However, HPV-negative HNSCCs commonly occur in the oral cavity, larynx, and hypopharynx [26]. Moreover, roughly 90% of oral cavity HNSCCs are HPV-negative, whereas about 70% of oropharyngeal HNSCCs are HPV-positive. Altogether, it is assumed that nearly 25% of HNSCCs are associated with high-risk HPV types [27]. Unfortunately, the incidence of HPV-positive oropharyngeal cancer is increasing and has surpassed cervical cancer to become the most common HPV-related cancer in developed nations [28]. In the United States, the increasing occurrence of HPV-positive oropharyngeal cancer mainly affects young white individuals (less than 60 years) and the male population (males-62% vs. females-56%) [29,30].

Different studies have shown a higher risk of head and neck cancers due to an increased number of sexual partners, practice of oral sex, and same-sex contact (i.e., MSM: men who have sex with men) [31,32]. In addition, evidence suggests an increased risk of oral HPV infection among MSM [33]. After examining data from the National Health Interview Survey, Behavioral Risk Factor Surveillance System, and National Youth Tobacco Survey, Kratzer and his colleagues observed a higher prevalence of smoking habits, obesity, and alcohol consumption among individuals within the LGBTQ+ community in comparison with heterosexual and cisgender people, suggesting an elevated risk of cancer [34]. Furthermore, Kratzer has clarified that many LGBT people experience discrimination even in the healthcare system, which can contribute to a higher prevalence of some cancer risk factors, although cancer incidence and mortality rates are not known accurately [35]. Regarding this issue, there is a scarcity of information on how oral health and levels of tobacco and alcohol use influence the risk [36]. It is worth mentioning that MSM have an elevated risk of anal cancer [37,38]. Another significant problem in MSM is lymphogranuloma venereum (LGV) proctocolitis that mimics inflammatory bowel disease and is caused by the L1–L3 serovars of *Chlamydia trachomatis* [39]. There has been an outbreak of LGV in industrialized countries since 2003. Studies have recorded a link between infection with *C. trachomatis* and high-risk HPV [40,41,42]. It is important to note that the number of people who self-identify as LGBTQ+ has risen in the past few years [43].

It has been reported that there is an increased risk of oropharyngeal cancer in men when their female partners have HPV-positive cervical intraepithelial neoplasia (CIN); however, no evidence of an increased risk of HPV-related lesions or cancer has been noticed among spouses/partners of patients with HPV-positive oropharyngeal cancer [27]. On the other hand, after analyzing the data from a few registries, including the Kentucky Cancer Registry, the authors documented that the incidences of both HPV-positive and HPV-negative cancers among smokers were higher for oropharyngeal and cervical cancers [44]. Of note, a number of cofactors are thought to be associated with cervical carcinogenic processes, such as smoking, long-term use of oral contraceptives, an unfavorable vaginal microbial environment, and nutrients or dietary factors [30]. The risk of both HPV-negative and HPV-positive oropharyngeal cancers are associated with cigarette smoking and alcohol consumption [2,29].

In HPV-positive oropharyngeal cancer, HPV-16 is associated with about 90–95% of cases, whereas HPV-18 accounts for roughly 3% of cases, and other high-risk HPVs are rarely detected [2,24,27,29]. By using HPV-16-positive oropharyngeal cancer-derived xenograft tissue, James et al. showed that about one-third of cases had integrated viral genomes (i.e., integrated into the host genome), another third had intact episomal HPV genomes with minimal integration, and the remaining third had either viral-viral episomes or viral-human DNA hybrids [45]. Of note, an episome is a plasmid-like structure that remains in the host cell without integrating into the eukaryotic genome. HPV-positive oropharyngeal cancer is known to originate from the tonsillar crypt epithelium, while HPV-negative cancer is likely to arise from the oropharyngeal surface epithelium [30,46]. Generally, HPV-positive cancer develops from the epithelium of the crypts in the palatine and lingual tonsils [45].

**Figure 1:**
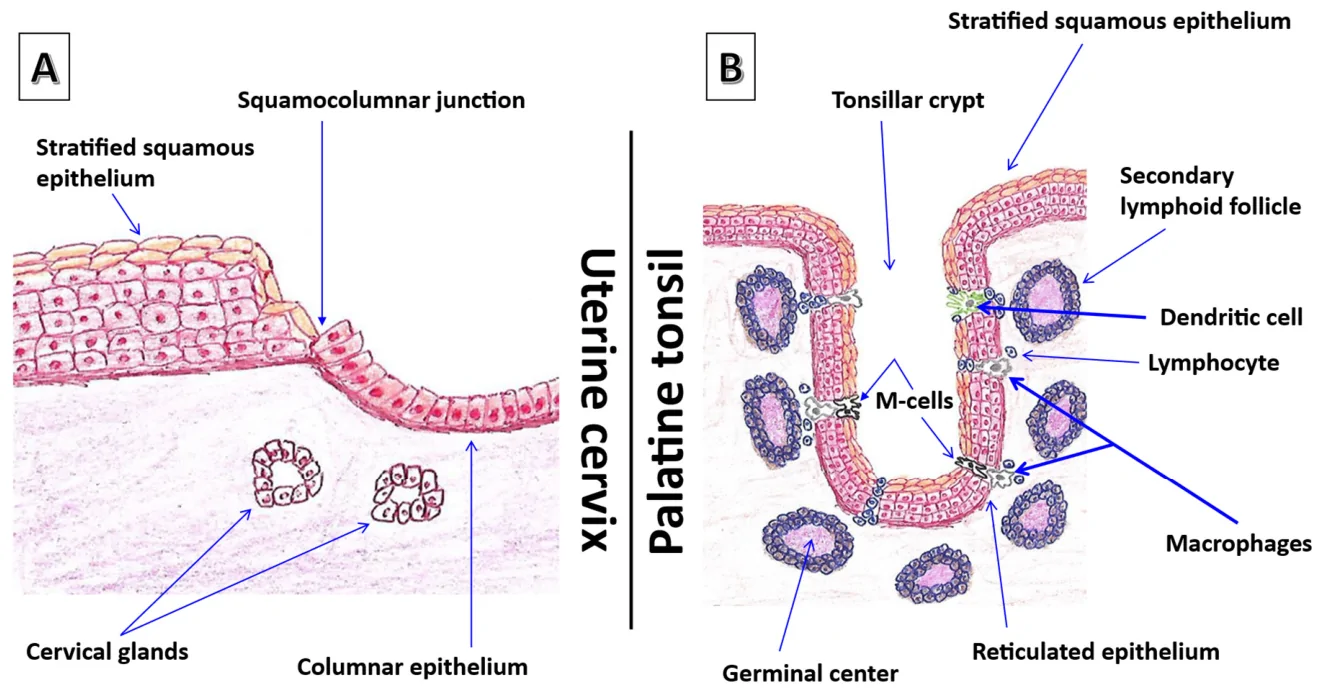
Schematic diagram of the cervical squamocolumnar junctional area (**A**) and a tonsillar crypt (**B**).

In the Waldeyer’s ring (encircling lymphoid tissue collections in the throat), there are four types of tonsils: palatine, lingual, tubal, and pharyngeal (adenoid) tonsils. The latter two structures, i.e., the tubal tonsil and the adenoid, are lined by pseudostratified ciliated columnar epithelium, whereas the palatine and lingual tonsils are lined by stratified squamous epithelium. The palatine tonsils have frequent crypts that are invaginations of the surface epithelium; crypt-like structures are also present in the lingual tonsils. Histologically, the squamous epithelium lining of the tonsillar crypts exhibits two distinct features: the coexistence of typical stratified squamous epithelium and reticulated epithelium, characterized by a loose, mesh-like organization that facilitates the presence of immune response cells, such as lymphocytes and M-cells [46,47,48]. It is believed that this loose or porous network can also facilitate HPV migration into the basal cells [49]. Regarding the carcinogenesis of the oropharynx, uterine cervix, and anal region, McBride logically concluded that inhabiting cells in transitional zones between different types of epithelia are specifically vulnerable [46]. Despite the fact that HPVs have tropism for squamous epithelium [50], the highest percentage of HPV-positive tumors (about 90%) originates from the cervical and anal regions [4]. However, tumor development in the anal region, along with other typical HPV-related sites, viz., vaginal, vulvar, and penile cancers, is rare, unlike cervical cancer (Table 1 [51,52,53,54]). Both anatomical sites, i.e., the cervical and anal regions, have a squamocolumnar junction. In anal carcinoma, the majority of tumors arise between the anorectal junction and the anal verge [55], which also includes the dentate/pectinate line (a squamocolumnar junction). On the other hand, in the uterine cervix, malignancies usually arise from the cervical squamocolumnar junction, where ectocervical squamous cells meet with the endocervical columnar cells or glandular epithelium (Figure 1). Throughout life, i.e., from the fetal stage to postmenopausal life, this junction shifts position due to several factors, including estrogenic influence, the acidic vaginal environment, and squamous metaplasia. It is interesting to note that the replacement of Müllerian columnar epithelium of the vaginal canal by stratified squamous epithelium occurs between 18 and 20 weeks of gestation [56]. Therefore, the cervical squamocolumnar junction is a dynamic area with high cellular activity, and the chances of errors during cell division (and hence cancer risk) could be higher, particularly in the presence of HPV’s E6 and E7. With regard to other HPV-related cancers, proportionally, 70% of oropharyngeal, vaginal, and vulvar cancers are HPV-positive, whereas 60% of penile cancer cases are HPV-positive [4]. The outer surface of all the abovementioned anatomical structures (i.e., vagina, vulva, and penis), where cancer develops, is lined by stratified squamous epithelium without any transitional area. Tonsillar crypts have a juxtaposition of two different types of squamous epithelia, and their tumors display a similar proportion of HPV-positive neoplastic growth to vaginal or vulvar cancers (i.e., ~70%, unlike cervical and anal cancers–around 90%). This phenomenon indicates a complicated pathological process of HPV-positive oropharyngeal cancer, which may be different from the well-studied cervical cancer [57].

Like HPV-positive cervical cancer, a better prognosis has been documented among patients with HPV-positive oropharyngeal cancer in comparison with HPV-negative cases [28,29,30,58]. However, there are currently no differences in treatment approaches between HPV-positive and HPV-negative oropharyngeal cancers. It is believed that HPV-negative oropharyngeal tumors have higher chromosomal alterations or genomic complexities than HPV-positive tumors [23,59]. In this regard, the frequently mentioned alteration is linked to p53, an important tumor suppressor and cell-cycle regulator. In HPV-negative cancer, there are p53 mutations, while p53 is often intact (but protein degradation) in HPV-positive cancer, which may influence the treatment outcomes [24,30]. Nonetheless, not all HPV-positive oropharyngeal tumors are similar; not all cases respond favorably [28]. The clinical course could be modified adversely by several factors, such as tobacco exposure, integration of HPV DNA into the host genome, elevated E6/E7 transcript levels, and higher pre-treatment circulating tumor DNAs.

Currently, immunohistochemical assessment of p16 is the only method (or the first step) to diagnose HPV-positive oropharyngeal squamous cell carcinoma. The immunohistochemical evaluation could preferably be followed by the detection of HPV DNA through either PCR or in situ hybridization. It is notable that pRb and p16 are tumor suppressor proteins, and they regulate the cell cycle, particularly the G1–S phase transition/checkpoint. The *CDKN_2_A* gene on chromosome 9 encodes p16 that inhibits cyclin-dependent kinases (CDK4 and CDK6), which essentially inactivate pRb by phosphorylation. In the situation of cell cycle arrest, pRb (unphosphorylated or hypophosphorylated) remains in complex with the transcription factor E2F. Phosphorylation of pRb causes the release of E2F, which is then capable of continuing the transcription of various genes, including *CDKN_2_A*, to resume the cell cycle. Therefore, pRb and p16 proteins are in a negative feedback loop: on the one hand, p16 inhibits pRb phosphorylation (leading to cell cycle arrest, including inhibition of p16 protein expression); on the other hand, pRb phosphorylation promotes p16 expression via E2F release. As mentioned earlier, E7 of HPV inactivates pRb (like phosphorylation), which allows cell cycle progression or uncontrolled cell proliferation, resulting in cancer risk, and overexpression of p16 in HPV-positive tumors. It is worth mentioning that p16 expression is often lost in most malignancies, including HPV-negative head and neck cancers, owing to gene mutation, deletion, or methylation [59].

Tonsillar crypts or mucosal invaginations possibly favor HPV capture, preservation, and support access of the virus to basal cells [24]. Because of the presence of virus DNA in the inaccessible location within the tonsillar crypts, the exact place of pathology may not be reached by the brush during sample collection [27,46]. For the abovementioned reasons, no precancerous lesions have been determined yet for oropharyngeal carcinoma, and no effective means of cancer screening has been formulated, although the frequency of this cancer is increasing in both males and females [60]. In contrast, screening programs for cervical cancer are well-established, including the identification of different intraepithelial or precursor lesions such as the presence of neoplastic lesions over the lower one-third of the epithelium (CIN1), two-thirds (CIN2), or the full thickness (CIN3) [46,60]. In this context, a general comparison between cervical cancer and oropharyngeal cancer has been shown in Table 2.

## 3. Cervical Cancer: Epidemiology and Diagnostic Approaches

An obvious socioeconomic scenario is observed in cervical cancer incidence and mortality. More than 85% of cases are recorded in developing countries [2]. In 2020 estimates, the highest age-standardized incidence rates were documented in different regions of Sub-Saharan Africa (i.e., Eastern Africa, followed by Southern Africa and Middle Africa) [61]. Like incidence rates, mortality rates also followed a comparable trend. The highest mortality rates were seen in Eastern Africa, and the lowest rates were noticed in Western Europe. Accordingly, high incidence rates were detected in countries like Eswatini (Swaziland), Malawi, and Zambia. In recent times, a decline in the incidence rates was observed in countries like Brazil, Colombia, and Costa Rica in Latin America; India, Thailand, and South Korea in Asia; and Poland, Slovenia, and Czechia in Europe [61]. This decline in developing countries can be attributed to several factors, including small family size (decreased fertility rate/parity), which is also connected to various aspects such as opportunities for proper education, associated lifestyle changes, and availability of adequate food and nutrition.

Regarding the risk factors of cervical cancer, different biological phenomena and social issues can impact the pathogenesis [62,63]. For example, pathogenesis has been thought to be linked with individuals’ specific sexual behavior, which includes multiple sexual partners, an early age of sexual activity, multigravida (three or more children), and use of oral contraceptives for a prolonged period (more than 5 years). Furthermore, an increased risk has been noticed in dysregulated immune conditions, such as human immunodeficiency virus (HIV) infection and systemic lupus erythematosus. Evidence also shows a higher risk for cervical cancer development in the pathologic alterations of cervicovaginal microbiota, e.g., a decreased relative abundance of beneficial *Lactobacillus* species, infection with *Chlamydia* or herpes simplex virus type-2 (HSV-2). Another important risk factor is cigarette smoking or tobacco use. Then again, an increased risk is associated with nutritional deficiencies that include vitamins A, C, D, and E, minerals like calcium, and different antioxidants. In connection with nutritional deficiencies, other related social issues, such as low socioeconomic status, limited educational opportunity, poor genital sanitation, and inferior access to medical care, have been documented to be associated with a higher risk.

In low- and middle-income nations, the mortality rate from cervical cancer is nearly 94%, and opportunities for screening and treatment are limited [63]. For the aforementioned socioeconomic circumstances, laboratory investigation procedures for HPV detection, which are routinely used in healthcare settings, may not be practically applicable. Some procedures that are useful in low- and middle-income countries are acetic acid solution test, Schiller’s iodine test, Pap test, and HPV DNA test (if possible). Cervical cancer screening programs in nations with appropriate medical and social resources have shown lower cervical cancer rates, suggesting that this preventive measure is able to decrease the incidence rate of cervical cancer [60].

**Figure 2:**
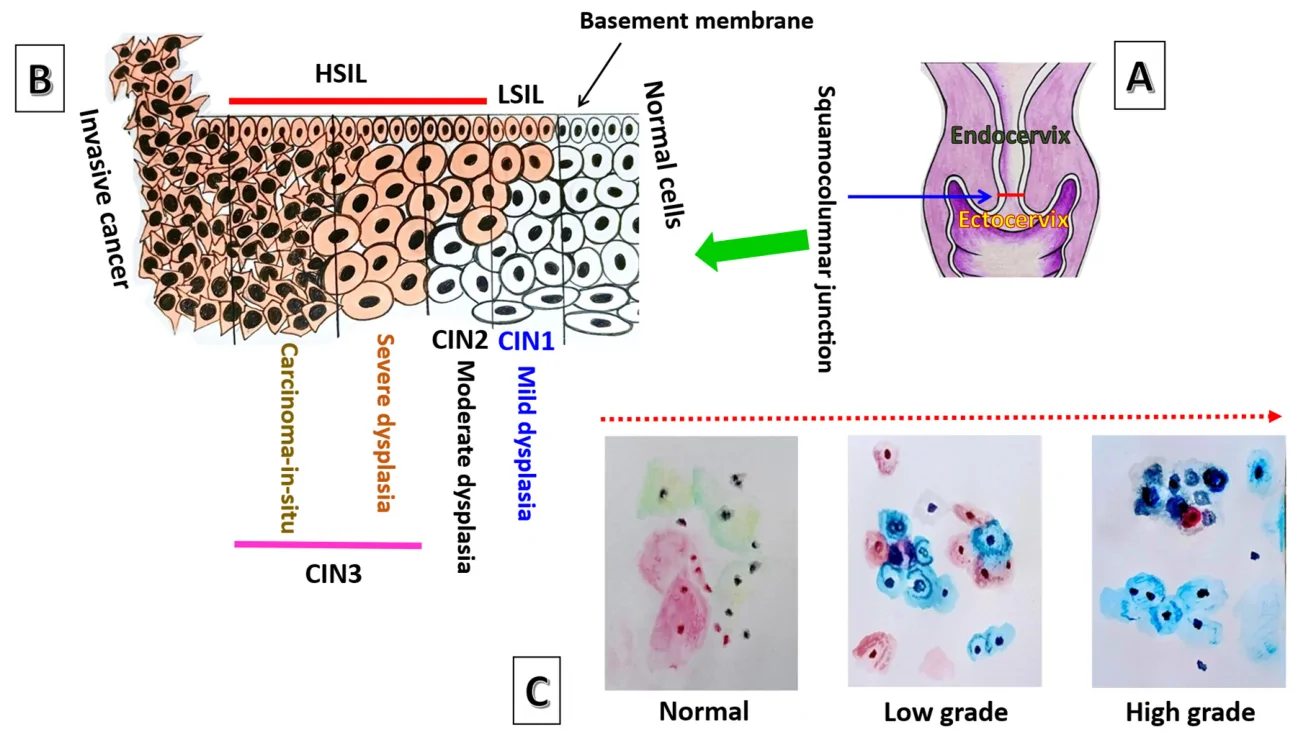
The natural history of cervical cancer. (**A**) Schematic diagram of the uterine cervix-endocervix, ectocervix, and the squamocolumnar junction. (**B**) Schematic diagram of the carcinogenic process–different stages of cervical lesions; LSIL: Low-grade squamous intraepithelial lesion; HSIL: High-grade squamous intraepithelial lesion; Cervical intraepithelial neoplasia–grade 1 (CIN1), grade 2 (CIN2), and grade 3 (CIN3). (**C**) Grades of dysplasia seen in Pap smear microscopy (illustrative representation). In normal cells, the cytoplasm appears much larger than the nucleus. Dysplastic cells have reduced cytoplasm and an increased nuclear-to-cytoplasmic ratio. Low-grade lesions are usually associated with HPV infections and may be resolved spontaneously. High-grade lesions are indicative of CIN (2 or more severe changes) or carcinoma-in situ.

In order to prevent cervical carcinogenesis or to detect pre-neoplastic lesions, there is a need to understand the natural history of cervical cancer. In the case of affected women, persistent HPV infection leads to a series of changes in cervical epithelium, ranging from mild dysplasia to pre-neoplastic conditions and finally malignancy (Figure 2). Generally, oncogenesis begins from the squamocolumnar junction of the uterine cervix and probably takes a decade or more to go through different precancerous states to finally become invasive cancer. For this reason, older women are generally at a higher risk, and for preventing the progression of these pathological processes, regular screening is certainly useful.

**Figure 3:**
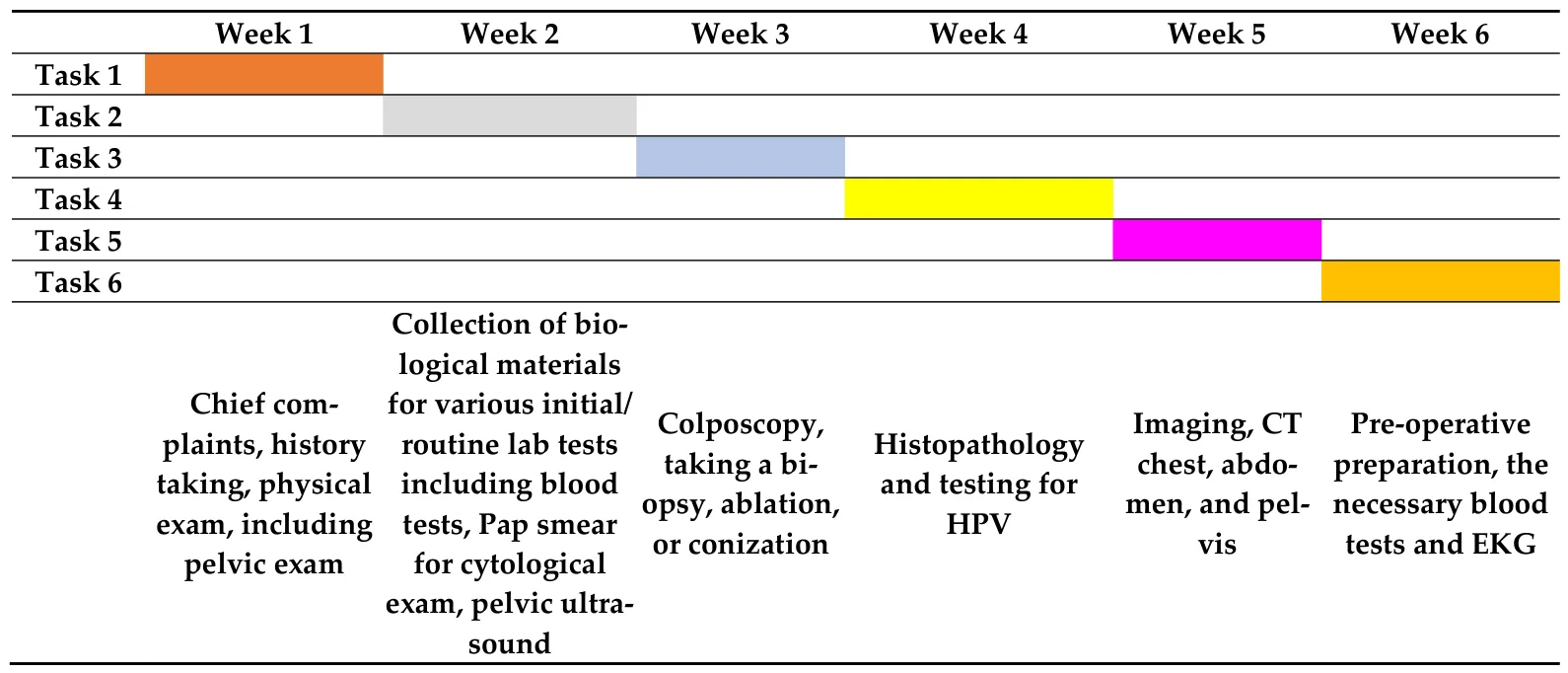
Management of cervical lesions/cancer; Illustrated as per the format of a Gantt chart and arranged the overall timeline based on the reports by Nanthamongkolkul & Hanprasertpong (2015) [67] and Hack et al. 2022 [68].

Regarding the indirect methods for detecting the presence of HPV, visual inspection of the uterine cervix after application of acetic acid (VIA) or Lugol’s iodine (Schiller’s test or VILI) has been employed, particularly in areas where advanced screening procedures such as colposcopy and cytopathology are unavailable. In the VIA test, dysplastic or neoplastic cells display an acetowhite appearance in the squamocolumnar junctional area as distinguished from normal cells [64]. Similarly, in VILI, iodine uptake is recorded as brown (positive uptake), yellow brown (partial uptake), as well as mustard yellow (no uptake), which is considered positive [65]. In positive cases, if the facility is available, biological samples (such as cervical biopsy and endocervical curettage) are collected for microscopic confirmation and HPV DNA detection. Nonetheless, with these two methods mentioned above, quick and inexpensive screening is possible, which also allows for initiating treatment without delay. Contrarily, these techniques are prone to subjective errors, and sometimes lesions can be difficult to detect for various reasons. It is believed that about 10% of HPV infections could be persistent, and roughly 3–4% develop intraepithelial lesions. If this neoplastic process continues, 0.7–1% may progress to high-grade lesions (i.e., CIN2 → CIN3), and ultimately, 0.1% of cases will advance towards invasive cancer [66]. This gradual progression of the lesion could be halted and even regressed to normal or satisfactory recovery by appropriate management in time (Figure 3 [67,68]).

Regarding the Pap test, it is a good method to examine morphological changes in a smear of cells collected from the squamocolumnar junction area. The technique was developed by George Papanicolaou in 1942; since then, there have been several modifications to this technique. Throughout the world, the technique has become instrumental in considerably reducing the prevalence of cervical cancer and accompanying mortality. Although it is not an expensive technique, the test evaluation is associated with subjective errors, and thus, professional expertise is required. Other important shortcomings of the Pap test include low sensitivity and specificity. A basic scheme of the Pap test has been described in Table 3 [69]. Along with a Pap smear, the HPV DNA test can be performed (namely, co-testing). The WHO guideline recommends DNA testing as a first-choice screening method for the detection of HPV and prevention of cervical cancer. Accurate HPV diagnosis is primarily dependent on the detection of HPV DNA from cervical samples (i.e., direct method for HPV detection).

Although the detection of HPV genomes by any method, such as dot blot, in situ hybridization, PCR, or hybrid capture, is generally beyond the financial capabilities of low-resource countries, the latter two methods exhibit high sensitivity and specificity. It is notable that several commercially available molecular diagnostic kits for HPV detection are currently used in a number of places [70]. Among these approaches, a commonly used technique is the hybrid capture assay system. The technique directly identifies the nucleic acid target and amplifies the chemiluminescent/fluorescent signal for detection, instead of PCR amplification of the target DNA. So, it utilizes the hybridization of nucleic acids and subsequent detection of chemiluminescence in the microplate. Sample HPV DNA is hybridized with an HPV-specific RNA probe, which produces a DNA:RNA hybrid molecule. The micro-wells are coated with the DNA:RNA hybrid-specific antibodies, which can capture the hybrid molecules. After the addition of alkaline phosphatase-conjugated antibodies (that bind the hybrid molecules), signal amplification occurs because multiple alkaline phosphatases are attached to individual antibodies, and many such antibodies can bind with every captured hybrid molecule.

## 4. The Role of Bacteria in Carcinogenic Processes

Many scientists believe that high-risk HPVs alone are not sufficient for cervical cancer development; the involvement of other factors is also necessary [30,71,72]. On this topic, Berman and Schiller commented that specific cervical microbial status is linked with the risk of high-grade lesions and cancer [2]. It is possible that almost all the risk factors can exert their adverse effects by unfavorably altering cervicovaginal (or vaginal) microbiota. For example, sexual behavior such as multiple sexual partners and multigravida can clearly change the vaginal microbial and associated biochemical characteristics. On the other hand, low socioeconomic status and nutritional deficiencies are usually linked, and these risk factors can impair immunity [73]. Although not exactly similar, a comparison can be drawn with the situation of HIV infection, which is related to alterations in both immunity and vaginal microbiota [74,75,76]. Studies also recorded the influence of various dietary components on vaginal microbial flora [77,78]. Conflicting evidence has been documented by different reports regarding the precise role of oral contraceptives [79,80,81,82,83]. However, smoking has been shown to affect the vaginal microbiota [84,85]. In a study in the Czech Republic, investigators analyzed samples from 250 women, and they found that smoking displayed an effect on the Nugent score, prevalence of clue cells (i.e., vaginal epithelial cells covered with bacteria), and growth of opportunistic pathogenic bacteria such as *Gardnerella* and *Mobiluncus* spp. [86]. Of note, the Nugent score system is used to diagnose bacterial vaginosis (i.e., an imbalance of vaginal bacteria ≈ dysbiosis) from vaginal smears on glass slides. At least 10 microscopic fields are inspected through oil-immersion for counting three categories of bacteria, namely *Lactobacillus*, *Gardnerella*, and curved Gram rods, and a score is achieved based on the number of counted bacteria using a 0 to 10 scale [87]. A score of 7 or more indicates bacterial vaginosis, which is more common in Black women. Nevertheless, another study in Brazil analyzed the 16S ribosomal RNA (16S rRNA) gene, which is used in bacterial identification, in vaginal samples from 609 women of reproductive age [88]. The investigators of this study noticed that smoking was independently associated with the community state type (CST) IV of vaginal microbiota. It is worth mentioning that, currently, the vaginal bacterial flora can be categorized into five bacterial CSTs or groups, depending on the abundance of beneficial *Lactobacillus* spp. [89]. Favorable *Lactobacillus*-dominated CSTs are CST I (*L. crispatus*), CST II (*L. gasseri*), and CST V (*L. jensenii*). However, women with *L. iners*-dominated CST III can acquire bacterial vaginosis. In contrast, CST IV is dominated by anaerobic bacteria such as *Gardnerella* and *Prevotella* and is commonly seen in patients with bacterial vaginosis as well as in healthy African women. This phenomenon could be an important reason for the abovementioned higher prevalence of bacterial vaginosis among Black women. Of note, many studies have observed a link between vaginal dysbiosis and cancerous lesions of the uterine cervix [90,91].

A *Lactobacillus*-depleted state and presence of a pro-inflammatory environment due to vaginal dysbiosis and relevant cytokines might have an active role in the development of CIN and eventually invasive cancer [92]. The squamocolumnar junction of the uterine cervix is a dynamic zone with high cellular activity, and a superimposed pro-inflammatory environment accelerates this process, and the resultant higher cellular turnover because of cell injury, repair or replacement by new cells in this chronic inflammatory condition can increase the errors in cell cycles, thus cancer risk, particularly in the presence of HPV E6 and E7 proteins [72]. Alternatively, it could be possible that, depending on the nature and duration of inflammation, the carcinogenic process might start without the presence of HPV, which perhaps is the cause of HPV-negative cervical cancer. It is known that chronic low-grade inflammation can promote malignant transformation, as seen in conditions such as obesity-related cancers, chronic parasitic irritation, and bacterial involvement in gastrointestinal malignancies [93,94,95]. Nevertheless, a study in Puerto Rico analyzed cervical samples of 91 participants from gynecology clinics [96]. The study observed that diminished *L. crispatus* was associated with higher concentrations of pro-inflammatory cytokines, along with a concomitant increase in dysbiosis-associated bacteria such as *Gardnerella*, *Prevotella*, and *Atopobium*. Similarly, in a study in China that included 210 subjects with high-risk HPV infection, the investigators noticed that the depletion of vaginal *Lactobacillus* was a significant predictor of CIN [97]. Another Chinese study examined 45 cases with cervical low-grade squamous intraepithelial lesions (LSIL), 36 high-grade lesions (HSIL), 27 cervical cancer cases, 22 HPV-positive cases without lesions, and 30 HPV-negative normal controls [98]. The study recorded a gradual depletion of *Lactobacillus*, particularly *L. crispatus*, which was related to the severity of cervical pathology. In the Dutch Cervical Cancer Screening Program, 492 high-risk HPV-positive and 500 HPV-negative cervical smears were analyzed, and it was found that the HPV-positive group had a significantly higher prevalence of *C. trachomatis*, *Mycoplasma genitalium*, and overall bacterial vaginosis as compared with the HPV-negative group [71]. Along the same lines, several investigators recorded that cervical neoplastic processes were associated with an abundance of a number of vaginal dysbiosis-related bacteria, e.g., *Atopobium*, *Gardnerella*, *Streptococcus*, *Ureaplasma urealyticum*, *Mycoplasma hominis*, and *C. trachomatis* [99,100,101].

Like cervical cancer, the oral microbiota probably plays an important role in head and neck cancer, including oropharyngeal cancer (Table 4 [8,102,103,104,105,106,107,108,109,110,111,112]). Virtually all studies on oral or head and neck cancers found microbial dysbiosis [113,114]. A recent document, after analyzing several reports, concluded that there was significant heterogeneity in the isolated bacterial taxa among different studies; although, overall, an increase in *Porphyromonas gingivalis*, *Tannerella forsythia*, and *Fusobacterium* was recorded in patients with oropharyngeal squamous cell carcinoma [115]. It is worth noting that malignancies of different organs derived from the primitive gut tube are linked with various bacteria, for instance, *Helicobacter pylori* in gastric cancer, *Chlamydia pneumoniae* in lung cancer, or *Salmonella typhi* in gallbladder cancer [116]. In addition, the large intestine is a classic example where active interaction between several factors, such as environmental elements, anomalous immune responses, and pathological alterations in microbiota (dysbiosis), can cause a range of different diseases, including inflammatory bowel disease and its associated complications, as well as colorectal cancer. Perhaps, a parallel situation exists in various head and neck cancers. It has been estimated that more than 700 bacterial species are present in the human oral cavity, and they play a crucial role in maintaining a healthy environment [117]. On the other hand, the prognosis of malignancies in the oral cavity is not satisfactory because a considerable number of cases are diagnosed at an advanced stage. Furthermore, it is noteworthy that 35–50% of the oral microbiota comprises unculturable species [118]. Therefore, the situation is somewhat challenging. However, DNA sequencing of these bacteria is gaining increasing attention. Currently, two different techniques are utilized for sequencing: the 16S rRNA gene sequencing, a commonly performed method, and the more recent approach of shotgun metagenomic sequencing. In general, the former technique involves sequencing 2–3 hypervariable regions, whereas the latter technique selects small DNA fragments randomly from the entire genome and independently sequences them.

In a recent study in Japan, biological samples (cancer tissue, saliva, dental and tongue plaque) were collected from 65 patients with HPV-negative oral and pharyngeal squamous cell carcinoma and 93 controls without cancer [117]. The investigators detected *Fusobacterium nucleatum* as the most prevailing bacterial species in cancer tissues. Notably, *F. nucleatum* is a Gram-negative anaerobic bacterium that can induce chronic inflammation and activate signaling pathways, including the Wnt/β-catenin and NF-κB. This activation results in the release of pro-inflammatory cytokines, such as interleukin-6 (IL-6), IL-8, and tumor necrosis factor-alpha, which contribute to the development of a chronic inflammatory condition and its associated sequelae [119]. Nocini et al. noticed that oropharyngeal cancer has a link with several bacteria, e.g., *Streptococcus anginosus*, *P. gingivalis*, and the genera *Capnocytophaga*, *Catonella*, *Dialisater*, *Filifactor*, and *Peptostreptococcus* [120]. Moreover, they believe that three specific bacteria, namely *Capnocytophaga gingivalis*, *Prevotella melaninogenica*, and *Streptococcus mitis*, could serve as salivary markers for the early detection of oropharyngeal cancer. A recent study in China examined samples from the dorsum of the tongue (n = 2017) and saliva (n = 1915); the study identified 13 microbial taxa, including *Prevotella*, *Neisseria*, *Veillonella*, and *Treponema*, which showed a causal relationship with oral and oropharyngeal squamous cell carcinoma [121]. In another study from the United States, oral rinse samples were collected for analysis of the microbial community from cases with squamous cell carcinoma of the oral cavity (n = 43), oropharynx (n = 64), and unknown primary sites (n = 5) before the initiation of cancer therapy, and control subjects (n = 242) [122]. Regarding microbial composition, the investigators observed differences between HPV-positive patients (n = 35) and HPV-negative patients (n = 55). In patients with HPV-positive cancers, members of the genera *Actinomyces*, *Campylobacter*, *Granulicatella*, and *Oribacterium*, as well as *Haemophilus parainfluenzae*, *Rothia mucilaginosa*, and *Veillonella dispar* were significantly increased, while *S. anginosus*, *Peptoniphilus*, and *Mycoplasma* were significantly reduced in comparison with HPV-negative patients. On the other hand, Powell and his colleagues mentioned a higher abundance of *Veillonella*, *Megasphaera*, and *Anaerolineae* in HPV-positive HNSCC compared to HPV-negative cases, which contrarily exhibited a dominance of *Haemophilus* [23].

The studies mentioned above usually fall under either cross-sectional or case–control designs. For understanding disease causation, these studies are less powerful than cohort studies [123]. However, cohort studies require substantial resources, which researchers working to establish novel ideas often lack. Therefore, findings from both cross-sectional and case–control studies are extremely helpful in initiating cohort studies to examine new ideas or hypotheses in greater depth. A similar situation can be expected here regarding the bacterial role in tumorigenesis.

Nevertheless, after invasive cancer develops and progresses, patients are at risk of several microbial infections, and bacterial composition also changes, differing from the normal microbiota. This pathological process could be associated with a range of factors such as underlying immune suppression, coexisting health problems, as well as poor nutritional and psychological issues, apart from complications related to therapeutic interventions. For example, head and neck cancers cause local mucosal injury and make patients susceptible to infections with pathogens such as streptococci, *Haemophilus influenzae*, and anaerobic bacteria [124]. Similarly, gynecological cancers damage barriers in the genitourinary tract, predisposing to infection with pathogens such as enterococci, enteric aerobic and anaerobic Gram-negative bacilli, and *Clostridium* spp. In addition, invasive cancers of certain sites, e.g., the head and neck, gastrointestinal, and genitourinary tracts, also predispose to infection with the flora inhabiting these sites [124].

The role of bacteria in various head and neck malignancies is significant, probably extending beyond their potential etiological connections. For example, surgery is an essential component in the management of head and neck malignancies, and a high rate of surgical site infection is an important cause of perioperative morbidity [125]. In general, cancer patients are more susceptible to secondary bacterial infections, and bacteria such as *Escherichia coli*, *Klebsiella* spp., and *Pseudomonas aeruginosa* are frequently isolated from patients with cancer [95,126,127]. In an in vivo study using the human oral squamous cell carcinoma cell line OSC-20 and BALB/c male mice, Woo et al. observed that *P. gingivalis*-infected cells exhibited higher resistance to the chemotherapy drug Taxol and a greater metastatic ability [128]. In another interesting study on oral squamous cell carcinoma cells, *F. nucleatum* was shown to promote partial-EMT (p-EMT) [129]. In this study, the investigators examined various oral cancer cell lines, such as HCS2, HOC621, HOC313, HOC719NE, and HOC719PE. Notably, EMT is a process in which epithelial cells acquire mesenchymal characteristics, which can be seen in a number of pathophysiological conditions, including cancer progression, and the intermediate state of this process is termed p-EMT (displaying both epithelial and mesenchymal features).

In microbial studies, in addition to challenges such as non-uniform methods of sample collection, transportation, storage, and DNA extraction, as well as the risk of contamination, laboratory methodological approaches also have their own issues [130]. Moreover, variability due to geographical location, social culture, interpersonal differences, and the effects of antibiotic use in different forms in society are important factors. Nevertheless, a complex, multidimensional network underlies microbiota-related pathogenesis. Current studies are generally limited to 16S rRNA or shotgun metagenomic sequencing. Although financial resources are a critical concern, integrating available approaches such as metagenomics, meta-transcriptomics, meta-proteomics, and metabolomics (i.e., multi-omics) is clearly preferable [130].

## 5. Lessons from Colon Cancer

A growing body of evidence suggests an important role of the intestinal microbial population in the development of colon cancer. For this reason, diverse microbial populations across different geographical regions may be linked to discrepancies in epidemiology and disease progression characteristics. Our gut microbiota consists of roughly 38 trillion bacteria, which are affected by various lifestyle-related factors, such as obesity, high-fat diets, and red meat consumption, leading to inflammation and abnormal cellular growth [131,132]. An increase in the incidence of early-onset colon cancer has been noticed since the beginning of the 1990s [133]. Nevertheless, alongside the processes of tumorigenesis, several reports have documented the prevalence of a number of bacteria, for example, *E. coli*, *Bacteroides fragilis*, *F. nucleatum*, and *Clostridium difficile* [131,132,133]. These bacteria may influence tumorigenesis by releasing bacterial metabolites and toxins/genotoxins that can cause DNA damage and alter immune responses and inflammation. Intriguingly, bacterial genotoxins can reactivate persistent viruses, such as the JC virus and the Epstein–Barr virus, which are implicated in cancer risk [134]. It is worth noting that the aforementioned bacteria may be involved in colon tumorigenesis through various mechanisms [133,135]. For instance, the genotoxin colibactin, produced by pks^+^ *E. coli* (having the polyketide synthase genomic island) can cause DNA damage. Toxin (i.e., BFT) released from *B. fragilis* can cause E-cadherin cleavage, stimulation of c-myc, and activation of Wnt/β-catenin and NF-κB. As mentioned earlier, the virulence factors of *F. nucleatum* (basically a member of the oral microbiota) can induce inflammation and EMT signaling. Furthermore, *C. difficile* toxin TcdB can promote Wnt signaling, reactive oxygen species, and DNA damage.

In a recent study in China, investigators analyzed colonic tissue-resident microbiota from 1134 clinical samples, including normal mucosa, precancerous polyps, and cancer cases, and observed microbial alterations as the clinical condition progressed from normal mucosa to invasive carcinoma [136]. This study documented a growing abundance of bacteria believed to have pro-tumorigenic roles, such as *Fusobacterium*, *Streptococcus*, and *Peptostreptococcus*, alongside a reduction in protective commensals such as *Collinsella* and *Akkermansia*. Another study on colorectal polyps (n = 30, 86.7% tubular adenomas) reported a relative abundance of *Bacteroides*, *Fusobacteria*, and *Proteobacteria*, along with a decline in *Bifidobacterium* in the polyp group, whereas the control group showed a relative abundance of *Actinobacteria* [137]. On the other hand, Hill et al. examined tumor and normal colorectal tissues from a group of participants (n = 898) in the Seattle Colon Cancer Family Registry for *F. nucleatum*, which was found to be consistently higher in tumor tissue samples [138]. Furthermore, another study by Queen et al. on colorectal cancer in Malaysian patients revealed a striking enrichment of *F. nucleatum* in tumors (97/112, 86.6%), particularly in cases with biofilms [139]. Of note, biofilm formation within the gut, which is adherent to the mucosal surface, promotes bacterial survival and inflammatory changes that may contribute to carcinogenesis. These investigators also observed significant enrichment of *B. fragilis* in the tumor microbiota and relative depletion of *Alistipes onderdonkii*, *Bifidobacterium longum*, *Parabacteroides distasonis*, and *Ruminococcus bromii*. In a retrospective cohort study that included 100,181 individuals, the overall positivity rate for *C. difficile* was 17%; however, recurrently positive individuals had a significantly elevated risk of incident colorectal cancer in comparison to those who were positive only once or never [140].

In recent years, the beneficial role of commensal and probiotic bacteria, including well-known *Lactobacillus* and *Bifidobacterium* spp., in inflammatory bowel conditions and colon cancer has been reported in several in vitro and in vivo studies. These bacteria may contribute significantly to preventing chronic inflammation and carcinogenesis by maintaining immune homeostasis, epithelial integrity, and the production of bioactive metabolites such as short-chain fatty acids (SCFAs) [141]. In addition, a lower level of *Lactobacillus* and *Bifidobacterium* is often documented in patients with colon cancer compared to healthy controls. It is thought that *Bifidobacterium* spp., such as *B. longum*, *B. adolescentis*, and *B. bifidum*, can prevent the growth of colon cancer cells by various mechanisms, including suppression of EGFR, HER2, and COX-2 expression levels [142]. By using both in vitro and in vivo experiments, Silva de Oliveira and colleagues showed that exopolysaccharides from *B. pseudolongum* and *B. pseudocatenulatum* promoted anti-tumor immunity [143]. In a study on azomethanes/dextran sodium sulfate (AOM/DSS)-induced C57BL/6J female mice, Niechcial et al. observed that *Bifidobacterium* can reduce colonic inflammation and prevent tumor development [144]. Of note, AOM/DSS is used to develop inflammation-associated colon cancer. Nevertheless, in another study, pre-treatment with a probiotic cocktail containing *B. longum*, *Lactobacillus acidophilus*, and *Enterococcus faecalis* showed favorable anti-cancer effects, increased *Lactobacillus* dominance, and reduced levels of pathogenic bacteria such as *Parasutterella*, *Helicobacter*, and *Muribaculum* in AOM/DSS-induced male C57BL/6 mice [145]. Similarly, administration of *Parabacteroides johnsonii* in AOM/DSS-induced C57BL/6 mice has been shown to decrease inflammatory levels, delay tumor growth, and improve the composition of the intestinal flora, including an increase in the abundance of *B. pseudolongum* and *Lactobacillus* [146]. Although these probiotic-related studies are currently in the preclinical stage, the use of the beneficial microbiota could be a promising strategy for cancer prevention and therapy.

## 6. The Gut–Vaginal Axis

The oral microbiota possibly affects both the local and distal portions of the alimentary tract. Locally, alterations in microbial composition and diversity not only affect the pathological processes of HNSCC; certain oral bacteria, such as *F. nucleatum*, *Porphyromonas gingivalis*, and *Olsenella*, could also increase the risk of colon cancer [147,148,149]. Similarly, the gut microbiota may influence cervicovaginal microbiota. Since cervical cancer is a major public health problem worldwide, the discourse would be limited to this topic only. A recent study in China examined samples of vaginal and anal swabs from patients with cervical cancer (n = 23), CIN1 (n = 62), CIN2 (n = 123), CIN3 (n = 51), and normal controls (n = 89) [150]. The investigators observed distinct patterns in the intestinal microbiota associated with different HPV infections, including enrichment of *Allisonella* in the HPV-16 group and a higher prevalence of *Lactobacillus*, *Staphylococcus*, and *Sellimonas* in the HPV-58 group. Another study on 72 cervical cancer patients (HPV-negative cases–27.8%) and 11 healthy controls documented differences in the composition and diversity of the vaginal and gut microflora between cancer cases and control women [151]. To investigate the vaginal and gut microbial characteristics during cervical carcinogenesis, Han et al. analyzed 416 vaginal samples (84 cervical cancer, 152 CIN, and 180 normal) and 116 gut samples (65 cervical cancer and 51 normal) [152]. The study noticed significant differences in vaginal and gut microbes in cervical cancer patients in comparison with the non-cancerous group. For example, *Firmicutes* were dominant in vaginal and gut microbial communities from non-cancerous women, whereas *Firmicutes* were significantly diminished in patients with cervical cancer. In a cross-sectional study in Mexico, fecal specimens and peripheral blood samples were collected from 49 cervical cancer patients (27 newly diagnosed/pre-treatment group and 22 patients- after-treatment) and 28 healthy controls for gut microbiota profiling [153]. Patients with cervical cancer displayed significant gut dysbiosis and enhancement of pro-inflammatory taxa such as *Escherichia*-*Shigella* and *Prevotella*. In addition, reduced patient survival was associated with dysbiosis and an apparent exhausted phenotype of natural killer cells. Consistent with the aforementioned trend, Zhou et al. observed that alterations in the gut and genital microbiota were associated with various gynecological diseases, including bacterial vaginosis and cervical cancer [154]. In their systematic review and meta-analysis, 87 studies out of 3327 articles were examined, and one of the most consistent changes was the enrichment of *Prevotella* and the depletion of *Lactobacillus* in these diseases.

It is believed that the adult human gut microbiota comprises roughly 10^13^–10^14^ cells (i.e., ten trillion to one hundred trillion), and that there are more than 1000 bacterial species, which largely inhabit the colon [155,156]. Similarly, the vaginal region is inhabited by trillions of bacteria, thought to originate from the gut microbiota. Nevertheless, in the gut, two phyla, namely *Bacteroidetes* and *Firmicutes*, constitute about 90% of the bacterial population. It is worth noting that dysbiosis, or microbial imbalance, is associated with an increase in the *Firmicutes*/*Bacteroidetes* ratio, a decrease in bacterial diversity, and an increase in the abundance of anaerobes [157]. Dysbiosis reduces β-glucuronidase activity, which is normally expressed by gut bacteria and deconjugates estrogens into the active forms. Several bacteria, including *Bifidobacterium*, *Lactobacillus*, and *Clostridium*, are involved in this process [158]. In gut dysbiosis, deconjugation is reduced, resulting in lower circulating estrogen levels [157]. The decline in estrogen levels reduces *Lactobacillus* abundance, thereby increasing vaginal alkalinity and promoting the growth of anaerobic bacteria in the vaginal environment [157,158]. Menopause is also associated with the lowering of estrogen levels and *Lactobacillus* depletion; the abovementioned situation can support bacterial vaginosis and thus enhance cancer risk.

Microbial translocation between the anorectal region and the female genitourinary tract, due to anatomical proximity, is perhaps a common phenomenon [158,159,160]. For example, these two sites share over 30% of bacterial species, and to some extent, bacteria in the gut possibly serve as a reservoir for the bacterial population in the vagina [160]. The vaginal *Lactobacilli* are believed to originate from the gut [155,156]. It is also hypothesized that IgA supports the migration of *Lactobacilli* from the ileum and their colonization in the vagina [161]. On the other hand, the dominance of *Prevotella* in both rectal and genital regions has been shown to be linked with an elevated risk of genital inflammation [159]. In general, both sites, i.e., the gut and vagina, have been demonstrated to harbor 5 common bacterial phyla, viz., *Bacteroidetes*, *Firmicutes*, *Fusobacteria*, *Actinobacteria*, and *Proteobacteria* [156]. Notably, the transfer of different bacterial strains from the gastrointestinal tract to the female genital tract may also occur via the hematogenous route [155,156].

Therefore, the gut microbiota can influence the vaginal microbial community both directly (by relocating bacterial species) and indirectly (via estrogen). Moreover, the gut microbiota functions as an important regulator of immune homeostasis [162]. For instance, adequate levels of SCFAs are produced by a healthy gut microbiota, and these SCFAs, upon entering circulation, activate immune regulatory pathways and appropriately modulate immune cells such as macrophages, dendritic cells, and T cells. Clearly, this immune homeostasis is disturbed in gut microbiota dysbiosis. Overall, a growing body of evidence suggests a significant role for the gut microbiota in both normal and pathologic conditions across different tissues/organs [159]. For this reason, various gut-organ axes, which are bidirectional communication systems, have been hypothesized, e.g., the gut–brain axis, gut–heart axis, gut–lung axis, and gut–skin axis, or broader interactions that include the gut–metabolic and gut–immune axes [163]. However, unlike the aforesaid biological communication systems or axes, the oral–gut and gut–liver axes have a direct anatomical connection or continuity, while the gut-vagina axis has an anatomical contiguity.

In a recently published review, the authors enumerated sites influenced by the gut microbiome in oncogenesis, including oral squamous cell carcinoma, colorectal cancer, and cervical cancer [164]. Moreover, they identified common microbiome-related oncogenic mechanisms, including pro-inflammatory signaling, DNA damage induced by bacterial toxins, and bacterial metabolite-associated cancer progression. Given the important role of *Lactobacillus* abundance in the vaginal microbiota, several authors have proposed new approaches, such as using probiotics to restore the healthy vaginal microbiota, thereby clearing HPV infections, reducing inflammation, and modulating immune responses [165,166,167]. In fact, a number of studies have shown significant effectiveness of probiotics in improving cervicovaginal wellness (Table 5 [168,169,170,171,172,173,174,175,176,177]). Probiotics can be administered orally or topically, although this form of microbial intervention has not yet been standardized. However, an appropriate understanding of the functional role of probiotics could be beneficial to develop novel management strategies in the prevention of several disorders of the female genital tract, including cervical cancer.

## 7. Conclusions

In cervical cancer pathogenesis, many scientists believe that HPV alone is inadequate to cause lesions. Perhaps HPV requires some other risk factor(s) to initiate this pathological process. Furthermore, among these risk factors for cervical carcinogenesis, almost every risk factor has been demonstrated to change the vaginal microbiota unfavorably. An alteration of vaginal microbiota and resultant chronic low-grade inflammation persistently supports the pathological processes of dysbiosis and accompanying complications, including the carcinogenic process. Overall, microbiota alterations are associated with HPV persistence and disease progression. On the other hand, a similar situation may happen in HPV-positive oropharyngeal cancer due to certain sexual behavior. More investigative work needs to be done to establish causality and further clinical utilization. However, establishing or restoration of favorable microbial flora could be an important strategy for prevention. It is worth mentioning that scientists are considering using vaccine along with probiotics to improve the effectiveness of cancer preventive or therapeutic strategies [178]. In this regard, a recent Japanese study is notable, where an attenuated HPV-16 E7-expressing *Lactobacillus casei* was given orally in a placebo-controlled randomized trial in patients with CIN2 lesions and HPV16 infection [179]. However, the investigators in this study did not observe significant differences in clinical and immunological parameters among participants. Currently, studies on probiotics on different cancers are generally cross-sectional or case–control studies, but the results of these early studies should help initiate appropriate cohort studies with a representative sample population.

## Figures and Tables

**Table 1:** Aproximate assessment of incidence rates and ranking of different cancers that are usually categorized as HPV-linked malignancies.

Cancer Site	Information		Global Ranking			In the United States		
	No.	Source	Among Women	Among Men	Total/Including All Cancers	Among Women	Among Men	Total/Including All Cancers
Cervical cancer	1	WHO	4th, Incidence: ~660,000 (in 2022)	–		–	–	–
	2	WCRF	4th		8th			
	3	CDC (2025)	–	–		Incidence: ~13,000	–	–
	4	HPV Information Centre	–	–	–	14th	–	–
	5	Weir et al. 2021 [51]						20th
Vaginal cancer (a rare cancer)	1	GCO†	–	–	35th, Incidence: ~19,000	–	–	–
	2	WCRF	27th	–	–	–	–	–
	3	CDC (2025)	–	–	–	Incidence: ~900 (75% HPV+)	–	–
	4	ACS				1–2%		
	5	Florida Department of Health *	–	–	–	–	–	0.3% of incident cases
Vulvar cancer (a rare cancer)	1	GCO†	–	–	31st, Incidence: ~47,340	–	–	–
	2	WCRF	21st	–	–	–	–	–
	3	CDC (2025)	–	–	–	Incidence: ~4500 (69% HPV+)	–	–
	4	Stehman & Look, 2006 [52]	–	–	–	5–8%	–	–
	5	NCI	–	–	–	–	–	0.4% of all new cases
Anal cancer (a rare cancer)	1	GCO	–	–	30th, Incidence: ~54,300	–	–	–
	2	Islami et al. 2017 [53]	Incidence: ~14,500 (in 2008)	Incidence: ~12,500 (in 2008)	–	–	–	–
	3	CDC (2026)	–	–	–	Incidence: ~5700 (93% HPV+) **	Incidence: ~2600 (89% HPV+) **	Incidence: ~8300 **
	4	NCI						0.5% of all new cases
Penile cancer (a rare cancer)	1	GCO†	–	–	32nd, Incidence: ~37,700	–	–	–
	2	WCRF	–	24th	–	–	–	–
	3	CDC (2025)	–	–	–	–	Incidence: ~1400 (63% HPV+)	–
	4	ACS	–	–	–	–	–	Less than 1% of cancers
Oropharyngeal cancer	1	GCO	–	–	24th, Incidence: ~106,400	–	–	–
	2	WCRF	26th, Incidence: ~20,100 (in 2022)	18th, Incidence: ~86,340 (in 2022)	–	–	–	–
	3	CDC (2026)	–	–	–	Incidence: ~3800 (63% HPV+)	Incidence: ~18,800 (72% HPV+)	Incidence: ~22,500
	4	Ellington et al. 2020 [54]	–	–	–	–	–	Oral cavity and pharynx cancers account for 3% of cancers

ACS: American Cancer Society, CDC: Centers for Disease Control and Prevention, GCO: Global Cancer Observatory, NCI: National Cancer Institute, WCRF: World Cancer Research Fund, WHO: World Health Organization; GCO†: Roughly estimated/slightly modified from the fact sheet of the Global Cancer Observatory; * Florida Department of Health–Annual Cancer Reports (Vagina, Vulva, and Other Rare Gynecological Cancers, 2004 Report); ** Includes rectal squamous cell carcinoma as well; NB: The information here shows the importance of the cervical squamocolumnar junction. Without it, the incidence rates of other HPV-related cancers worldwide are either moderate (such as oropharyngeal cancer) or in the rare-cancer category (such as vaginal, vulvar, and penile cancers).

**Table 2:** Different characteristics and dissimilarities between HPV-positive malignancies of the uterine cervix and oropharynx.

Features	Cervical Cancer	Oropharyngeal Cancer
Involved anatomical structure	The squamocolumnar junction of the uterine cervix	The crypts of the palatine and lingual tonsils
HPV’s involvement	Roughly 90% of total cases	Around 70% of cases with oropharyngeal cancer
HPV types	The majority are associated with HPV-16. Both HPV-16 and -18: about 70%. There is an increase in other types, such as HPV-45 and -52	The predominant type is HPV-16, accounting for more than 90% of cases
Patients	Generally, women from less developed nations	Men are affected more than women. Usually, young white males from developed nations
Major risk factors	Smoking, long-term oral contraceptive use, and nutritional deficiency	Oral sex, smoking, and alcohol consumption
Ideal preliminary diagnostic examination	Pap smear test	Immunohistochemistry of p16 protein
Detection of precancerous lesions and cancer screening	Well-defined precancerous lesions and cancer screening methods are properly planned	No precancerous lesions have been identified yet; therefore, no effective screening methods have been formulated
Overall management	Due to precise diagnostic and screening approaches, the course of action is clear	The management process is deficient due to a lack of an appropriate screening method
Prognosis: in relation to HPV-negative cases	Prognosis is better in HPV-positive cases compared to HPV-negative cases	Like HPV-positive cervical cancer, HPV-positive oropharyngeal cancer cases usually show a better prognosis compared to HPV-negative cases
Prognosis: cervical cancer vs. oropharyngeal cancer	Inferior, probably due to the poor socioeconomic status of the developing countries, which includes a lack of proper nutrition, as well as inadequate health screening and treatment facilities	Better, possibly due to the adjacent lymphoid structure, which is an important part of the immune system. Moreover, this cancer is prevalent in developed countries with appropriate access to healthcare facilities and nutrition
Vaccine efficacy	Maybe better, although it is not a perfect time to comment because of a prolonged latency period of cancer development	Perhaps not equivalent to the level of protection against cervical cancer

**Table 3:** Fundamental arrangement of the Pap staining technique.

Solutions	Components	Dyes	Characteristics
Solution 1	―	Hematoxylin	Basic nuclear stain
Solution 2	OG-6	―	Acidic cytoplasmic stain
		Orange G	Acid dye, stains keratin with a bright intense orange
		Phosphotungstic acid	Mordant, binds to the protein and intensifies the color
Solution 3	EA	―	―
		Light green SF	Acid dye, stains the cytoplasm of metabolically active cells: endocervical cells, parabasal cells, intermediate squamous cells, histiocytes, leukocytes, undifferentiated cancer cells, and adenocarcinoma cells as green
		Eosin Y	Acid dye, stains the cytoplasm of superficial squamous cells, nucleoli, erythrocytes and cilia
		Bismarck brown Y	Precipitates phosphotungstic acid, responsible for differential staining by Light green and Eosin

Pap staining method: After collecting the cells from the squamocolumnar junction, these cells are spread on glass slides and fixed. Fixation → 95% ethanol (15 s) → 80% ethanol (15 s) → 70% ethanol (15 s) → 50% ethanol (15 s) → 50% ethanol (15 s) → 0.5% HCl (1–2 dips) → Distilled water (10 dips) → Dist water (10 dips) → Harris hematoxylin (6 min) → Dist water (15 s) → Dist water (15 s) → Wash in tap water (15 s) → 50% ethanol (15 s) → 70% ethanol (15 s) → 80% ethanol (15 s) → 95% ethanol (10 dips) → OG-6 (2–3 min) → 95% ethanol (10 dips) → 95% ethanol (10 dips) → EA (2–3 min) → 95% ethanol (10 dips) → 95% ethanol (10 dips) → 100% ethanol (10 dips) → Alcohol xylene (1:1, 10 dips) → Xylene (10 dips) → Xylene (10 dips) → Mount with DPX. (Based on the information by Raju, 2016 [69]).

**Table 4:** Results of various studies recorded how different bacteria were associated with pre-neoplastic and neoplastic conditions of the uterine cervix and the head & neck regions, along with HPV.

Cancer Types	Study and Subjects’ Details	Findings
Cervical cancer	Chen X et al., 2026 [8]. The study included 10,104 Chinese women, 2207 (21.8%) were positive for HPV, of which the HR–HPV–positive were 1861 (84.3%).	The 3 most commonly detected HR-HPV subtypes were HPV-52, HPV-16, and HPV-58. Vaginal infections, including bacterial vaginosis, aerobic vaginitis, and *Trichomonas vaginalis*, demonstrated a higher chance of HPV infection.
	Chen Y et al., 2026 [102]. In this study in China, vaginal samples were analyzed from 102 women, who were classified into 4 groups: control group (HR-HPV-negative women, n = 26), HR-HPV-positive group (n = 22), HR-HPV-positive with mucositis (vaginal, n = 26), and CIN group (1, HR-HPV-positive, n = 28).	Compared with the control group, the HR-HPV-positive group showed increased abundances of *Bifidobacterium*, *Escherichia-Shigella*, and *Hoylesella*, along with a significant reduction in *Lactobacillus*. Compared with the HR-HPV-positive mucositis group, the CIN group displayed significantly lower *Pseudomonas* levels.
	Cho et al., 2026 [103]. Cervicovaginal samples from 72 vaccinated Korean women (20–35 years) were analyzed.	HR-HPV was present in 32 women (44.4%), the most common types: HPV-39 and -52. These women showed significantly higher microbial diversity, with bacteria such as *Gardnerella leopoldii*, *G. vaginalis*, *Megasphaera lornae*, and *Lactobacillus iners*. Women with abnormal cytology displayed significantly higher microbial diversity. *L. crispatus* was more common in the HPV-negative group.
	Gómez Cherey et al., 2026 [104]. A total of 741 Argentine women underwent clinical examination: 466 were HPV-negative, and HPV-16 was present in 82 cases.	HPV-16 was the most prevalent in HSIL/SSC and LSIL, and in the 18–50 age group. In the younger age group (18–24 years, n = 138), 54.2% of women with HPV-16 (13/24) had bacterial vaginosis. In the 25–50 years group (n = 456), 34.7% of women with HPV-16 (17/49) had bacterial vaginosis.
	Guo et al., 2025 [105]. This study in China examined cervical samples and vaginal secretions from 151 cases: HPV-negative (n = 51), transient infection (n = 42), and persistent infection (n = 58).	The microbial community included CST II (n = 5), CST III (n = 52), and CST IV (n = 94). Compared to HPV-negative women, cases with transient and persistent infections had lower vaginal cleanliness, higher pH, and fewer *Lactobacilli*. HPV infection was linked to vaginal microbiota affluence, with discrete group compositions. In persistent infection, *Sneathia amnii* was significantly elevated.
	Ji et al., 2026 [106]. Interferon-based local therapy was provided to 29 Chinese women with HR-HPV-associated LSIL or lower-grade cervical abnormalities and followed up for 3months.	The treatment failure or persistence group (n = 19) showed reduced *Lactobacillus* abundance but increased abundances of *Gardnerella*, *Streptococcus anginosus*, *Schaalia turicensis*, and *Comamonadaceae*. Whereas the clearance group (n = 10) exhibited the opposite: an increase in *Lactobacillus* and a decrease in *Gardnerella* and *Prevotella*.
	Li et al., 2026 [107]. The study enrolled 869 HR-HPV-positive cases and 473 negative controls in China.	The most prevalent pathogen was *Ureaplasma urealyticum* (45.75%), which was frequently detected in HR-HPV-positive women and was associated with an elevated risk of CIN1, whereas *Chlamydia trachomatis* was independently correlated with higher odds of ASC-US.
	Zhu et al., 2025 [108]. This study in China enrolled 76 patients: 32 with cancer (18 squamous cell carcinoma and 14 adenocarcinoma) and 44 with intraepithelial lesions (14 LSIL and 30 HSIL).	Compared with the intraepithelial lesions group, vaginal bacterial richness was significantly higher in the cancer group, with an increase in anaerobic bacteria such as *Prevotella* and *Sneathia*. There was a negative correlation between *Lactobacillus* abundance and anaerobic bacteria.
Head and neck cancer	De Martin et al., 2021 [109]. This Swiss study analyzed the microbiome of palatine tonsil crypts from patients with HPV-positive tonsillar cancer (n = 18) and tonsillar tissue from obstructive sleep apnea patients (OSA, controls, n = 14) who were negative for HPV. In the extended cohorts, there were 21 OSA–all were negative for HPV, and 28 tonsil tumor patients–3 were negative for HPV.	The investigators observed an increased abundance of the phyla *Firmicutes* and *Actinobacteria* in cancer patients. Moreover, cancer cases were associated with the accumulation of several genera such as *Veillonella*, *Streptococcus* and *Prevotella_7* in tonsillar crypts. In controls (OSA), *Spirochetes* and *Synergistetes* were significantly higher. *Fusobacterium*, *Prevotella* and *Treponema_2* were also enriched among controls. In extended cohorts, a significantly higher relative abundance of *Filifactor alocis* in crypt biopsies of OSA was found in comparison to patients with tumor.
	Kwak et al., 2024 [110]. A prospective nested case–control study based on 3 US cohorts-the American Cancer Society Cancer Prevention Study II Nutrition Cohort, the Prostate, Lung, Colorectal, and Ovarian Cancer Screening Trial, and the Southern Community Cohort Study. The study included 236 HNSCC cases (24.6% women), and 485 matched controls.	HPV-16 was found positive in 11 cases and 2 controls. For the risk of HNSCC development, the investigators identified 13 oral bacterial species, e.g., *Eikenella corrodens*, *Lactobacillus paracollinoides*, *Porphyromonas cangingivalis*, *Prevotella salivae*, *Pyramidobacter piscolens*, *Rodentibacter pneumotropicus*, *Simonsiella muelleri*, *Streptococcus gallolyticus*, *Pasteurella multocida*, *Streptococcus plurextorum*, and *Streptococcus sanguinis*.
	Maidment et al., 2025 * [111]. This Australian study of healthy individuals analyzed 128 oral samples: 64 were HPV-positive (27 HR-HPV), and 58 subjects were male.	HPV-negative men (n = 27) contained significantly higher abundance of *Streptococcus salivarius* than men with HR-HPV (n = 13). *Haemophilus parainfluenzae* and *Streptococcus sanguinis* were more abundant in HPV-negative women, whereas HPV-positive women (n = 32) had more abundance of *Neisseria subflava*.
	Upadhyay et al., 2024 [112]. A total of 499 samples from the Cancer Genome Atlas (TCGA) dataset were analyzed. Included primary tumors were from the oral cavity (60.7%), oropharynx (15.0%), hypopharynx (1.8%), and larynx (22.2%).	HPV-positive cases were 111; Alphapapillomavirus 9 (which includes HPV-16) was most commonly detected (90.1%), and its presence was associated with improved overall survival. Microbial species were more abundant in HPV-negative cases, which included *Citrobacter farmeri*, *Thermoanaerobacter kivui*, and *Yersinia pestis*. *Pseudomonas viridiflava* was associated with the best survival, whereas *Yersinia pseudotuberculosis* was correlated with the worst.

* Study on cancer-free subjects; ASC-US: atypical squamous cells of undetermined significance, CIN1: cervical intraepithelial neoplasia grade 1, CST: community state type, HNSCC: head and neck squamous cell carcinoma, HR-HPV: high-risk human papillomavirus, HSIL: high-grade squamous intraepithelial lesion, LSIL: low-grade squamous intraepithelial lesions, SCC: squamous cervical carcinoma.

**Table 5:** Selected recent studies that documented improvements in the vaginal microbial environment and associated clinical conditions, including HPV clearance after treatment with probiotics.

Authors, Place of Study, and Study Design	Findings/Outcomes
Bui et al. 2025 [168]; Vietnam | Vaginal-spray probiotic containing *Bacillus subtilis*, *B. clausii*, and *B. coagulans* spores was used among women with bacterial vaginosis. Assessment of vaginal microbiota was performed by day 7 (at the end of standard treatment) and day 28 (21 days post-treatment; control: 48 women and the same number in the probiotic group).	Compared with the control group, the probiotic group showed an accelerated decline in bacterial vaginosis symptoms. In the probiotic group, there was an increase in beneficial species such as *Lactobacillus crispatus* and *Streptococcus salivarius* as well as a reduction in harmful species such as *Gardnerella vaginalis* and *Sneathia sanguinegens*.
Di Pierro et al. 2025 [169]; Italy | Randomized, prospective, single-blind study in women with HR-HPV infection–probiotic group (n = 40) and placebo group (n = 22). Patients in the treated group used the probiotic strain *L. crispatus* M247 orally once a day for 4 continuous months. After completion of treatment, all participants in both groups underwent a PAP test, HPV-DNA test, colposcopy examination, and vaginal microbiota sampling.	Probiotic treatment significantly supports HPV clearance and negative PAP results. Moreover, the vaginal microbiota analysis showed a decreased richness, an elevated relative presence of *L. crispatus*, and a higher number of women with a CST I microbiota.
Filardo et al. 2024 [170]; Italy | This observational pilot study used oral capsules containing 4 strains of *Lactobacilli*, 3 strains of *Bifidobacteria*, and *Streptococcus thermophilus*, and were employed in asymptomatic bacterial vaginosis patients (n = 13) and women with healthy vaginal microbiota (n = 37). Participants were assessed at baseline and after 2 months of treatment using the Nugent score and metagenomic analysis.	In women with bacterial vaginosis, the diversity and abundance of the vaginal microbiota were significantly diminished. Some dysbiosis-linked bacteria such as *Megasphaera* spp. were significantly reduced, whereas *Lactobacillus* spp. increased.
Liu et al. 2024 [171]; China | Double-blind, placebo-controlled trial on patients with cervical HR-HPV infection (50 cases each in the probiotic group and the placebo group). The probiotic preparation contained *L. crispatus* chen-01 and was used vaginally: one capsule for 14 days in the first 3 months, then once every 3 days and 5 times a month in the 4th and 5th months. Cervical exfoliated cells were collected 6 months after for cytological and viral analyses.	Use of probiotic preparation effectively improved vaginal microbiota and significantly decreased HPV load, enhanced viral clearance, and ameliorated vaginal inflammation.
Nisaa et al. 2026 [172]; Malaysia | Randomized, double-blind, placebo-controlled clinical trial in healthy women evaluating the effectiveness of oral *Lactiplantibacillus plantarum* Probio87 for 12 weeks: probiotic group (n = 58) and placebo group (n = 54). At baseline and after 12 weeks, vaginal and fecal samples were analyzed by 16S rRNA sequencing.	In the probiotic group, there was a significant reduction in Nugent score. Microbiota analyses indicated that the probiotic strain preserved vaginal microbiota by controlling bacterial vaginosis-associated taxa and maintaining beneficial *Lactobacillales* (which include the genus *Lactobacillus*).
Rezazadeh et al. 2024 [173]; Iran | Patients with bacterial vaginosis were initially treated with metronidazole and then received beneficial *Lactobacillus* strains in capsules, administered either vaginally (n = 20) or orally (n = 35)–vaginally each night for 2 weeks and orally per day for 4 weeks. One month after completing the probiotic treatment, evaluation was done by the Nugent scoring system and assessed the presence of 3 bacterial types, i.e., *Lactobacilli*, *Gardnerella* and *Bacteroides*, as well as *Mobiluncus*.	In both groups, the Nugent score decreased to 3, suggesting the equivalent efficacy of both routes of probiotic administration. All patients exhibited significant improvements from their initial conditions.
Thekho et al. 2024 [174]; India | 60 patients with vulvovaginal candidiasis were divided into two groups–one group (n = 30) treated with oral antifungal fluconazole and another group treated with both oral fluconazole and oral probiotic capsules (*Lactobacillus rhamnosus*, *L. crispatus*, *L. gasseri*, *L. jensenii*) for 2 months.	A larger proportion of patients in the probiotic group (97%) achieved complete remission compared with the sole antifungal group (90%).
Vaccalluzzo et al. 2024 [175]; Italy | Randomized, controlled clinical trial that included 80 women with clinical features of vaginal dysbiosis. The treatment group (n = 60) took 1 capsule of the probiotic strain *Lacticaseibacillus rhamnosus* TOM 22.8 orally for 10 consecutive days. Evaluations such as Amsel criteria, Nugent score, and Lactobacillary grade were performed at baseline, 10 days, and 30 days after the treatment.	Compared to the non-treatment group, administration of the probiotic strain increased the *Lactobacilli* population in the vaginal environment and decreased the population of potentially pathogenic microorganisms. This resulted in restored physiological pH and reduced clinical problems.
Yang et al. 2026 [176]; China | Prospective study on women with persistent HR-HPV infection–participants were divided into 3 groups: (1) intervention group (n = 124) received probiotic *Enterococcus faecium* vaginal capsules (10 days as one treatment course, maximum 3 treatment courses/3 months) and anti-infective treatment in case of vaginitis, (2) regular follow-up group (n = 120) received treatment for vaginitis, without probiotic, (3) in the blank control group (n = 121), participants were examined at 0 and 6 months after the study initiation and treatment received in case of vaginitis. Participants’ HR-HPV status and microecological parameters were evaluated at 0, 6, and 12 months.	Probiotic supplementation resulted in a significantly higher HPV clearance, a marked increase in the abundance of *Lactobacillus*, and a significant decline in several pathobionts, such as *Atopobium*, *Prevotella*, and *Staphylococcus*.
Zhang et al. 2025 [177]; China | Randomized, controlled trial on patients with asymptomatic bacterial vaginosis, who received either a 7-day course of oral metronidazole (n = 358) or 10-day intravaginal probiotic capsules (*Lactobacillus delbrueckii* subsp. lactis DM8909, n = 358).	At the end of the 1st, 2nd, and 4th week after the completion of therapy, the cumulative cure rates were higher in the probiotic group. At the end of the 2nd, 3rd, and 4th months, cured women were followed up; recurrence rates were 13.2% (19/144) in the probiotics group and 56.1% (69/123) in the metronidazole group at the end of the 4th month. The difference in cumulative recurrence rates between these 2 groups was statistically significant.

CST: Community state type; HR-HPV: High-risk human papillomavirus; Amsel criteria: Clinical diagnosis of bacterial vaginosis depends on characteristics of vaginal discharge, vaginal pH (more than 4.5), fishy odor after adding 10% potassium hydroxide to vaginal fluid, and microscopic detection of clue cells (bacteria-coated vaginal epithelial cells). Lactobacillary grade: Microscopic assessment of the quantity and proportion of protective *Lactobacilli* in the vaginal fluid: the score ranges from I (normal) to III (abnormal). Metagenomic analysis: Examination of the entire genetic material of all microorganisms in a natural habitat. Nugent score: Microscopic scoring system by assessing large Gram-positive rods (*Lactobacillus*), small Gram-variable rods (*Gardnerella vaginalis*/*Bacteroides*), and curved Gram-variable rods (*Mobiluncus* spp.) in a vaginal sample.

## Data Availability

No new data were created or analyzed in this study. Data sharing is not applicable to this article.
